# Preclinical safety testing of an affinity-optimised MAGE-A10 T cell receptor for adoptive T cell therapy

**DOI:** 10.1186/2051-1426-3-S2-P14

**Published:** 2015-11-04

**Authors:** Andrew Gerry, Joseph Sanderson, Manoj Saini, Barbara Tavano, Roslin Docta, Nicholas Pumphrey, Miguel Maroto, Ellen Border, Alan Bennett, Gwendolyn Binder-Scholl, Tom Holdich, Lini Pandite, Rafael Amado, Bent Jakobsen

**Affiliations:** 1 Adaptimmune, Abingdon, PA, USA; 2 Adaptimmune, Abingdon, UK; 3 Adaptimmune, Philadelphia, PA, USA

## Background

Preclinical safety testing for adoptive immunotherapies utilising T cell receptor-(TCR) engineered T cells relies on stringent target validation and a robust pre-clinical specificity testing strategy. Specific safety concerns include on-target off-tumour toxicity and off-target toxicity. Avoidance of these toxicities requires that the expression profile of the target antigen be shown to be specific to tumour tissues, and that the TCR is shown to be specific.

## Methods

We evaluated the cancer testis antigen, MAGE-A10 (melanoma antigen family A10). Mage-A10 was found to be expressed in multiple tumour samples including lung [all indications] (16.4%, 23/140), lung squamous cell carcinoma (30.4%, 14/46) and breast (2.2%, 3/135). Mage-A10 RNA expression was measured across 72 normal tissues (3 samples each), and no expression was detected outside of placenta and very low expression was detected in testis, an immune privileged site. Thus, we concluded Mage-A10 was an attractive tumour target.

## Results

21 parental TCRs specific for the selected HLA-A2 restricted Mage-A10 peptide, were identified; three were selected for affinity enhancement. Fifteen affinity-enhanced TCRs were screened in cell based assays against a panel of tumour cells lines +/- for HLA-A2 and/or Mage-A10 expression, resulting in the selection of two candidates based on potency and specificity in ELISpot assays. To establish the fine specificity of each TCR, a peptide binding motif was established, using a panel of 172 peptides representing substitution of each amino acid position with all other possible amino acids, and searched against the human genome. Mage-A10 clone 796 (Mage-A10^c796^) was selected based on this molecular mapping, which demonstrated only 3 other peptides that the TCR could potentially recognize; none were subsequently recognized in cell based assays, thus demonstrating the specificity of Mage-A10^c796^.T cells expressing Mage-A10^c796^ (Mage-A10^c796^–T) were screened against 59 HLA-A2 positive normal cells and 22 MAGE-A10 negative tumour cell lines by IFN-γ ELISpot, and no off-target reactivity was revealed. Potential alloreactivity of Mage-A10^c796^–T was tested using a panel of 67 B cell lines expressing 129 different HLA alleles. Alloreactivity against HLA-B*1501 and B*4601 was identified. These alleles will form part of exclusion criteria for clinical trials. Additional assays performed to assess potential cytotoxicity and cytokine release all demonstrated good on-target potency with no identified on- or off-target safety concerns.

## Conclusions

These data support the clinical application of Mage-A10^c796^–T. The IND is now open and a dose escalation study in lung cancer is due to start in the second half of 2015.

